# A Rare Finding of Incidental Intracholecystic Papillary Neoplasm Following Acute Cholecystitis Management

**DOI:** 10.7759/cureus.41222

**Published:** 2023-06-30

**Authors:** Sara Arfan, Kapish Sharma, Lavanyah Anbazhagan, Timothy J Stear

**Affiliations:** 1 Department of Surgery, Windsor University School of Medicine, Cayon, KNA; 2 Department of Surgery, Community First Medical Center, Chicago, USA

**Keywords:** gallbladder papillomatosis, gallbladder wall thickening, laparoscopic cholecystectomy (lc), acute calculus cholecystitis, intracholecystic papillary tubular neoplasm

## Abstract

Intracholecystic papillary neoplasm (ICPN) is a grossly visible, mass-forming, noninvasive epithelial neoplasm arising from the mucosa and projecting into the lumen of the gallbladder. ICPN is a lesser-known tumor of the gallbladder lining, which although has a better prognosis compared to gallbladder adenocarcinoma carries the potential for metastatic transformation with spread to other organs. ICPN is found incidentally on imaging or during postop histological evaluation. However, we present a unique case of ICPN that was incidentally diagnosed in a 72-year-old Eastern European woman following cholecystectomy for acute cholecystitis and was missed on preoperative imaging. Follow-up protocols of ICPN are poorly understood and vastly underreported. We discuss this patient’s findings in light of current literary evidence available on ICPN and outline future directions for better clinical understanding. We also highlight the need for screening guidelines in light of known risk factors to better understand the natural history of the disease to prevent malignant transformation into invasive gallbladder carcinoma.

## Introduction

Intracholecystic papillary neoplasm (ICPN) is a premalignant lesion arising from the gallbladder lining [1]. ICPN is a potentially fatal neoplasm that can progress to invasive gallbladder carcinoma if left untreated [1]. ICPN was initially described in 2012, and its classification, identification, and natural history of the disease remain largely unknown [2]. To date, 123 cases were studied, and three microscopic growth patterns (tubular, papillary, and tubulopapillary) and four morphologic patterns (biliary, gastric, intestinal, and oncotic) were identified [2]. Based on current statistical data, a higher rate of ICPN development is seen in women compared to men, with a ratio of 2:1 [2]. ICPN demonstrates architectural similarities and a similar clinical progression to other metastatic gastrointestinal tumors. These include intraductal papillary neoplasm (IPN) located in the bile ducts, intra-ampullary papillary-tubular neoplasm (IAPN) located in the ampulla, and intraductal papillary mucinous neoplasms (IPMN) as well as intraductal tubulopapillary neoplasms (ITPN) located in the pancreas [2,3]. ICPN commonly occurs in the body or fundus of the gallbladder [3] and classically presents with right upper quadrant (RUQ) pain. It is detected incidentally through conventional imaging techniques such as ultrasound (U/S), computed tomography (CT), and magnetic resonance imaging (MRI) [4]. Although premalignant and clinically asymptomatic, once the lesion progresses beyond the gallbladder, treatment is complicated by lymph node dissection and adjacent chemotherapy, instead of cholecystectomy [5]. We present the case of a 72-year-old Eastern European woman with ICPN that was missed on preoperative imaging and was incidentally discovered upon pathological analysis post-cholecystectomy, a phenomenon that is witnessed in <1% of ICPN cases [2].

## Case presentation

A 72-year-old Eastern European woman presented to the emergency department (ED) complaining of mid-epigastric and RUQ abdominal pain radiating to the back for the past three days. The pain was not associated with the consumption of meals and was diffuse on presentation. The patient also reported nausea and vomiting for the past three days and constipation for five days. Her past medical history was remarkable for diabetes mellitus and hypertension. The RUQ pain had gradual onset, was intermittent in nature, and was rated at an 8 out of 10 in severity.

Hemodynamic parameters on admission included a temperature of 98.6°F, a pulse of 104 breaths per minute (bpm), respiratory rate of 18 breaths per minute (bpm), blood pressure of 142/82 mmHg, and oxygen saturation of 96% on room air. A review of the systems was positive for diffuse abdominal pain, nausea, vomiting, and constipation. On physical exam, the abdomen was soft but tender to palpation in both mid-epigastric and RUQ regions. Bowel sounds were normal, and there was no distension, masses, rigidity, guarding, or costovertebral angle (CVA) tenderness. Murphy’s sign was positive.

Laboratory results revealed a normal white blood cell count (WBC) of 7.5 K/uL (reference range: 5-10 K/uL), an elevated red blood cell count (RBC) of 5.31 x 1012/L (reference range: 3.8-5.2 x 1012/L), a normal hemoglobin (Hb) of 16.0 g/dL (reference range: 12-16 g/dL), a normal hematocrit (Hct) of 46.4% (reference range: 36%-48%), elevated serum glucose of 377 mg/dL (reference range: 70-100 mg/dL), a decreased serum sodium level of 130 mmol/L (136-145 mmol/L), a decreased serum chloride of 92 mmol/L (reference range: 96-106 mmol/L), and a normal prothrombin time (PT) of 12.6 seconds (11-13.5 seconds). Liver function testing demonstrated a decreased aspartate aminotransferase (AST) level of 5.0 U/L (reference range: 8-33 U/L), a normal alanine transaminase (ALT) level of 29.0 U/L (reference range: 4-36 U/L), and a normal alkaline phosphatase level of 56 IU/L (44-147 U/L).

**Figure 1:**
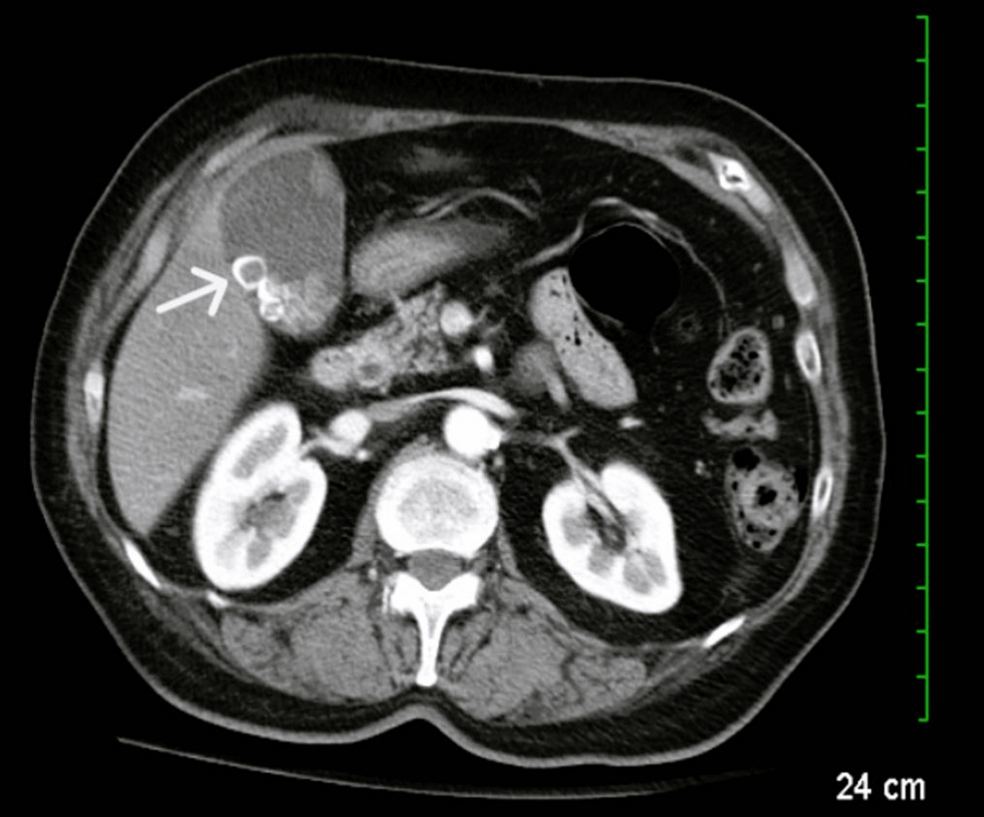
Abdominal CT demonstrating cholelithiasis (white arrow) CT: Computed tomography.

A CT of the abdomen and pelvis with intravenous (IV) contrast revealed a distended gallbladder with numerous gallstones, consistent with acute cholecystitis (Figure 1).

**Figure 2:**
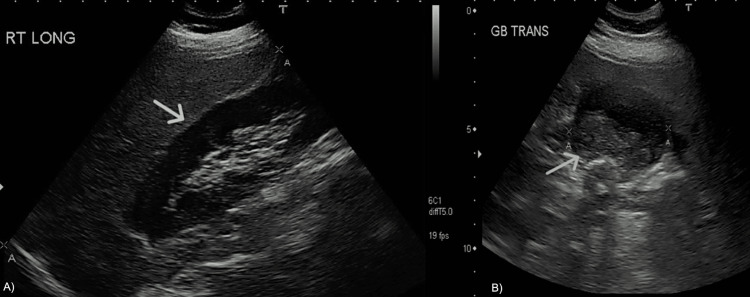
Right upper quadrant ultrasound demonstrating cholelithiasis with gallbladder distention (white arrow, Panel A) in the longitudinal plane and biliary sludge (yellow arrow, Panel B) in the transverse plane

There was also fatty infiltration of the liver. Imaging was negative for intra- or extra-hepatic ductal dilation. A RUQ U/S revealed gallbladder distension, cholelithiasis (Figure 2, Panel A), and biliary sludge (Figure 2, Panel B).

**Figure 3:**
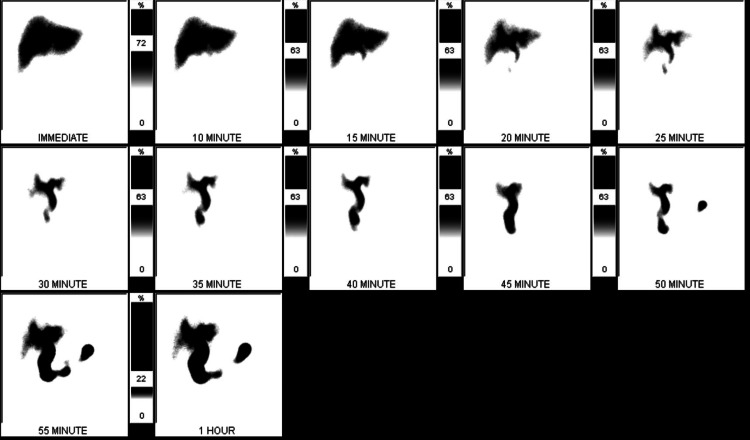
Hepatobiliary scan with nonvisualization of the gallbladder and small bowel activity noted at 25 minutes

A hepatobiliary scan confirmed absent scintigraphic activity in the gallbladder with 6.1 mebrofenin choletec injection (mCi) technetium-99m mebrofenin, which was consistent with cholecystitis (Figure 3).

The patient was diagnosed with acute cholecystitis and underwent laparoscopic cholecystectomy. The procedure was tolerated well. On postoperative day one, the patient was tachycardic with a rate of 112 bpm but did not have any complaints otherwise. She was discharged on postoperative day three in stable condition.

**Figure 4:**
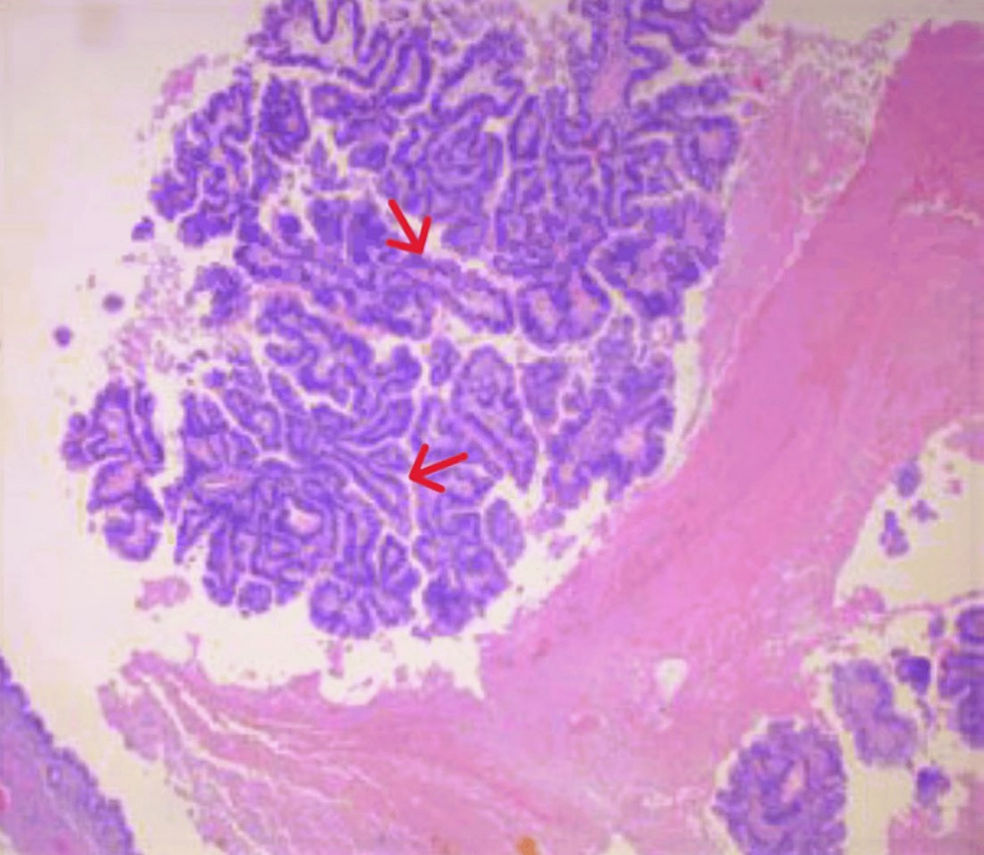
Microscopic findings of intracholecystic papillary neoplasm, demonstrating high papillary architecture along thin fibrovascular stalks (hematoxylin and eosin staining; magnification ×400)

The patient was diagnosed with acute cholecystitis and underwent laparoscopic cholecystectomy. The procedure was tolerated well, and the resected gallbladder specimen was sent to pathology. On histopathologic exam, the gallbladder was filled with thick dark green bile and measured at 9.0 x 4.0 x 3.5 cm and was 0.3 cm in thickness. Further evaluation revealed a rough, polypoid cheese-like lesion at the fundus, measuring 3.0 x 2.0 cm. The cystic duct margin was free of the lesion and was negative for high-grade dysplasia and malignancy. The histopathological evaluation confirmed that the lesion was an intracholecystic papillary neoplasm (Figure 4). Additionally, multiple brownstones were identified, measuring 0.3-0.9 cm on average. The stones were fragmented and had an orange crystalized interior.

On postoperative day one, the patient was tachycardic with a rate of 112 bpm but did not have any complaints otherwise. She was discharged on postoperative day three in stable condition. On postoperative day nine, the patient presented with right flank pain radiating down to her leg. She also reported feeling a “lump” in the RUQ accompanied by pain with meals, which improves a few hours after meals. Abdominal U/S revealed an absence of biloma or any post-surgical complication. Due to the lack of postop surgical complications, the patient was readmitted to the medical floor on postop day nine and was not followed up by the surgical team.

## Discussion

This case highlights the presentation of acute cholecystitis in an atypical age demographic with a non-classical presentation and incidental discovery of ICPN. Typically, patients who present with acute cholecystitis are in the third to fifth decade of life and are predominantly women. Our patient presented in the seventh decade of life, without fever or leukocytosis, and without post-prandial RUQ pain. Diagnostic imaging via abdominal U/S and IV contrast CT confirmed the presence of cholelithiasis with increased thickening of the gallbladder wall but no identifiable mass.

ICPN is a rather uncommon premalignant neoplasm. Typically, it presents as an intermittent RUQ pain with a detectable mass (≥1 cm) on conventional imaging such as U/S or contrast CT. Up to 50% of ICPN cases are detected incidentally, and approximately 10% are missed in imaging studies [6]. Due to the rare occurrence of this lesion, current literature has only identified a few risk factors that may lead to the development of this neoplasm. These include advanced age in the seventh and eighth decades of life and a higher disposition toward the female gender. Other risk factors include a history of gallstone disease, which have a higher prevalence in patients with ICPN compared to the general population. Lastly, Eastern Asian populations and chronic inflammatory states also contribute to a higher risk of ICPN development [7].

The diagnostic protocol of ICPN involves a combination of in-depth clinical evaluation, imaging studies, and histopathological analysis. Diagnostic steps involve clinical evaluation where a thorough medical history and physical examination are conducted, and an assessment of the patients’ symptoms, risk factors, and other medical comorbidities is conducted. U/S, CT, and MRI are the current standards of imaging for detecting ICPN lesions. U/S detects the presence of gallbladder polyps/masses and assesses the size, location, and appearance. CT reveals information about the extent of the tumor invasion into nearby structures and distant or lymph node metastasis. MRI, on the other hand, provides further information about tumor invasion and differentiates a benign lesion from a malignant one [6]. Contrast-enhanced endoscopic ultrasound (CEUS) and endoscopic retrograde cholangiopancreatography (ERCP) are performed to evaluate the biliary tree and check for obstruction or involvement of other organs caused by tumor invasion [7]. Lastly, histopathological analysis and tissue sampling are performed under imaging guidance. Free needle aspiration (FNA) biopsy also differentiates benign and malignant lesions. The classic treatment of ICPN involves surgical resection in the form of cholecystectomy [1]. The resected gallbladder is then sent for histopathological examination for the assessment of tumor stage and grade [1].

ICPN, along with its counterparts (IPN, IAPN, IPMN, and ITPN), follow the adenoma-carcinoma sequence with mutations such as KRAS, STK11, CTNNB1, and APC (adenomatous polyposis coli) being commonplace [8,9]. ICPN displays a wide range of histopathological features that help further classify its subtype and help establish a deeper understanding of each type and its associated prognostic outcomes. The major histopathological features that help distinguish subtypes are microscopic growth patterns, grade of dysplasia, and morphological patterns. Microscopic patterns seen with ICPN are papillary, tubular, and tubulopapillary and are applied based on the dominant microscopic growth pattern. The growth pattern associated with the highest rate of malignant transformation is papillary, which is also the most common pattern (>75%) [6]. As seen with other premalignant lesions, ICPN is also graded as low-grade dysplasia, high-grade dysplasia, or invasive carcinoma [10]. Morphological patterns seen with ICPN are biliary (most common), gastric (subclassified into foveolar and pyloric), intestinal, and oncocytic phenotypes [10]. Histopathologic features that have shown statistical correlation with malignant transformation are papillary microscopic growth patterns, biliary or foveolar phenotype, and the presence of high-grade dysplasia [10].

Although most cases of ICPN are asymptomatic, it is a premalignant lesion with the propensity to evolve into invasive carcinoma in 6.4% of the cases [6]. Despite malignant transformation accounting for a minor percentage of invasive carcinoma, this case presentation demonstrates how readily these lesions may evade radiological detection. If undetected, due to its asymptomatic nature and a lack of screening guidelines despite known risk factors, transformation into high-grade invasive carcinoma leads to adverse patient outcomes. Thus, the importance of early detection cannot be understated. Due to the rare occurrence of ICPN, there is a scarcity of data to establish improved diagnostic procedures and screening guidelines. Our main intent in reporting this case is to bring awareness to this rare and potentially fatal neoplasm.

## Conclusions

ICPN is a rare neoplasm that can be difficult to detect on conventional imaging modalities. Although rare, a small fraction can evolve into invasive carcinoma. Our case illustrates how often these rare lesions can go undiagnosed due to the lack of surveillance and imaging protocols. This unique incidental case of ICPN adds valuable information to the existing literature as the disease onset and progression are unexpected and confirms recent findings, which highlight the use of CEUS and MRI to be superior in detecting ICPN compared to the traditional conventional U/S and CT imaging. The lack of surveillance and diagnostic protocols can lead to delayed diagnosis at advanced stages of malignancy with a poor prognosis compared to patients diagnosed earlier and treated with prompt surgical removal. We hope that our contribution to the existing database will aid in raising clinician awareness of ICPN and related neoplasms as a diagnosis to screen for in patients who may be at the risk of developing it.
